# Sirt1 Deficiency Attenuates Spermatogenesis and Germ Cell Function

**DOI:** 10.1371/journal.pone.0001571

**Published:** 2008-02-13

**Authors:** Matthew Coussens, John G. Maresh, Ryuzo Yanagimachi, Gregg Maeda, Richard Allsopp

**Affiliations:** 1 John A. Burns School of Medicine, University of Hawaii, Honolulu, Hawaii; 2 Institute for Biogenesis Research, University of Hawaii, Honolulu, Hawaii; Ordway Research Institute, United States of America

## Abstract

In mammals, Sirt1, a member of the sirtuin family of proteins, functions as a nicotinamide adenine dinucleotide-dependent protein deactylase, and has important physiological roles, including the regulation of glucose metabolism, cell survival, and mitochondrial respiration. The initial investigations of Sirt1 deficient mice have revealed a phenotype that includes a reduced lifespan, small size, and an increased frequency of abnormal sperm. We have now performed a detailed analysis of the molecular and functional effects of Sirt1 deficiency in the germ line of Sirt1 knock-out (−/−) mice. We find that Sirt1 deficiency markedly attenuates spermatogenesis, but not oogenesis. Numbers of mature sperm and spermatogenic precursors, as early as d15.5 of development, are significantly reduced (∼2-10-fold less; P≤0.004) in numbers in Sirt1−/− mice, whereas Sirt1 deficiency did not effect the efficiency oocyte production following superovulation of female mice. Furthermore, the proportion of mature sperm with elevated DNA damage (∼7.5% of total epididymal sperm; P = 0.02) was significantly increased in adult Sirt1−/− males. Analysis of global gene expression by microarray analysis in Sirt1 deficient testis revealed dysregulated expression of 85 genes, which were enriched (P<0.05) for genes involved in spermatogenesis and protein sumoylation. To assess the function of Sirt1 deficient germ cells, we compared the efficiency of generating embryos and viable offspring in *in vitro* fertilization (IVF) experiments using gametes from Sirt1−/− and sibling Sirt1+/− mice. While viable animals were derived in both Sirt1−/− X wild type and Sirt1−/− X Sirt1−/− crosses, the efficiency of producing both 2-cell zygotes and viable offspring was diminished when IVF was performed with Sirt1−/− sperm and/or oocytes. Together, these data support an important role for Sirt1 in spermatogenesis, including spermatogenic stem cells, as well as germ cell function.

## Introduction

The *Sir* (Silent Information Regulator) genes were originally discovered in yeast (Saccharomyces cerevisiae) where they were shown to affect expression of genes, due to their ability to promote heterochromatin, at the HML and HMR mating type loci [1]. In lower eukaryotes, Sir2 functions as an NAD-dependent histone de-acetylase [2] and has been shown to have pro-longevity effects [3]. Sirt1, a member of the mammalian Sirtuin gene family, is the best candidate for an ortholog to the yeast Sir2 gene [4], [5] and is capable of de-acetylating a number of protein substrates including, but not limited to, p53 [6] and FOXO transcription factors [7].

Recent studies have demonstrated that Sirt1 has a number of important physiological roles in mammals. Most notably, it plays an important role in the regulation of glucose metabolism. Sirt1 has been shown to promote gluconeogenesis in the liver [8] and increase respiration and insulin secretion in pancreatic β-cells [9]. The former process involves deacetylation of FOXO1 and PGC1-α by Sirt1 [8] whereas the latter process involves Sirt1-dependent suppression of expression of uncoupling protein 2 (UCP2) which uncouples respiration from ATP production in the mitochondria [9]. In addition, Sirt1 has been shown to protect against oxidative stress in β-cells, thru a process involving deacetylation of FOXO proteins [10]. Furthermore, Sirt1 promotes fat mobilization and inhibits fat cell differentiation in white adipose tissue via a mechanism involving interaction of Sirt1 with peroxisome-proliferator-activated receptor-γ (PPARγ) and suppression of the transcriptional activity of PPARγ [11]. It has been suggested that these and other affects of Sirt1 in mammalian cells may act to counter metabolic syndrome, the dysregulation of glucose homeostasis, which becomes more pronounced with age in some mammals including humans [12]. Sirt1 has also been shown to have pro-survival effects on neurons. For example, the neuroprotective affect of the mutation in Wallerian degeneration slow (*Wld^s^*) mice has been shown to be dependent on SirT1 [13]. Moreover, Sirt1 over-expression has been shown to protect against β-amyloid induced death of microglia by a mechanism involving inhibition of NF-KB signaling [14]. While the exact nature of the neuro-protective effects of Sirt1 on neurons is presently unknown, it likely involve inhibition of apoptosis by the interaction of Sirt1 with a number of different proteins involved in the survival response to stress, including p53 [6] FOXO transcription factors [7] NF-KB[15], E2F1 [16], and Ku70 [17].

Sirt1 deficient (knock-out) mouse strains have now been developed in a number of independent studies [18]–[20]. In all strains, Sirt1 deficient mice have a small, feeble phenotype, with significantly increased post-natal mortality rates. Sirt1 deficient mice also display a number of developmental defects, most noticeably, irregular shaped eyes, evident in embryos as early as d16.5 [19] with adult mice often failing to open one or both eyelids [18], [19] and defective cardiac septation [19]. Initial analysis of the germ line in Sirt1 deficient mice has shown marked attenuation in development of the testis, and elevated numbers of abnormal sperm in adult male mice [18]. However, in mouse embryonic fibroblasts derived from Sirt1 knock-out mice, Sirt1 deficiency actually promotes extension of replicative lifespan, and survival following exposure to genotoxic stress [21]. Together these data suggest that the effect of Sirt1 deficiency is pleiotropic and dependent on cell type and/or stage of development.

In the present study, we have performed a detailed analysis of the effect of Sirt1 deficiency on the function of male and female germ cells at the cellular and molecular level. We show that the low number of spermatozoa and atrophied testis in male Sirt1 deficient mice is due to effects that manifest at least as early as d15.5 of development, and correspond with elevated frequency of DNA damage in mature sperm in adult mice. Microarray analysis of global gene expression in testis from Sirt1 deficient mice reveals aberrant expression of a number of genes that have important roles in spermatogenesis, as well as genes involved in sumoylation. Furthermore, using in vitro fertilization (IVF), we show that both male and female gametes from Sirt1 deficient mice have a reduced efficiency at generating viable zygotes, although these zygotes are fully capable of developing to term following embryo transfer to pseudo-pregnant females.

## Materials and Methods

### Mice

The Sirt1 knock-out (−/−) strain [19] and Oct4-GFP transgenic mouse strain [22] were kindly provided by Fred Alt (Harvard University) and Dr. Jeff Mann (University of Melbourne) respectively. To generate Sirt1−/− embryos for primitive spermatogenic stem cell analysis, Sirt1+/− mice were bred with Oct4-GFP mice and then backcrossed to generate Sirt1+/− mice that were homozygous for the Oct4-GFP transgene, which were then bred together. The mice were fed with a standard diet and maintained in a temperature and light-controlled room (22°C, 14L:10D; light starting at 0700 h), in accordance with the guidelines of the Laboratory Animal Services at the University of Hawaii and the Committee on Care and Use of Laboratory Animals of the Institute of Laboratory Resources National Research Council (DHEW publication 80–23, revised in 1985).

### Sperm Count

For analysis of epididymal sperm, male mice were sacrificed using cervical dislocation and the caudal epididymis was dissected out and cut. Epididymal sperm was extracted using forceps and placed in a droplet of HTF media overlayed with mineral oil. After capacitation, an aliquot near the top of the droplet was removed, and sperm were counted using a hemocytometer.

### Analysis of Primitive Spermatogenic Stem Cells

To assess the effect of Sirt1 deficiency on early spermatogenic stem cells, we bred Sirt1+/− mice homozygous for the Oct4-GFP transgene to generate Sirt−/−,Oct4-GFP embryos (see above), which are easily distinguished from Sirt1+/−,Oct4-GFP or Sirt1+/+,Oct4-GFP embryos on the basis of size (the Sirt1 deficient embryos are much smaller; genotype of all embryos was also confirmed retrospectively by PCR). Gonads were then isolated from d15.5 male embryos, and single cell suspensions for FACS analysis of stem cell numbers were generated as previously described [23].

### Histology

Tissues were removed from mice and fixed in Bouin's solution overnight. The following morning, samples were washed in 70% ethanol, dehydrated, and embedded in paraffin. Six-micrometer sections were prepared on glass slides, cleared in xylenes, and stained with fresh hematoxylin for 10 minutes, washed, and then immediately stained with eosin. Following staining, slides were dehydrated in ethanol, air-dried, then mounted in balsam for microscopic analysis.

### In Vitro Fertilization

In vitro fertilization (IVF) experiments were performed using established protocols [24], [25]. Superovulation was induced in female mice by administration of 5IU of PMSG followed by 5IU of hCG 48–52 hours later. Oocytes were harvested 14 hours post hCG injection, placed in 200ul of HTF media overlayed with mineral oil, and incubated at 37C [24]. Sperm was harvested from the cauda epididymis of male donor mice using forceps, placed in 300ul of HTF media, and incubated for 30 minutes to induce capacitation. Capacitated sperm was then co-incubated with the mature oocytes for 2–3 hours. The oocytes were subsequently transferred and washed through several drops of CZB media and incubated overnight at 37C. The next morning, 2-cell embryos were counted and transferred to fresh CZB. Embryos were either transferred into oviducts of pseudopregnant CD-1 females later in the evening, or incubated for an additional 3 days in CZB media to assess efficiency of blastocyst development.

### Embryo Transfer

Following IVF, surrogate mothers were prepared by mating female CD-1 mice in estrus with vasectomized CD-1 male mice. Twenty-four hours later, the pseudopregnant CD-1 females were anesthetized and 2-cell embryos were injected into each oviduct and allowed to develop to term.

### Comet Assay

Epididymal sperm was isolated and re-suspended in HTF media. Each sperm sample was then promptly pelleted, re-suspended in low melt agarose at 37C, and applied as a uniform layer on glass slides. Alkaline comet assays were performed according to the manufacturers' protocol (Trevigen, Gathersburg, MD) with minor revisions. Specifically, 40 mM DTT was added to the lysis solution during initial incubation, without Proteinase K, for one hour. Slides were then further incubated in lysis solution containing 10ug/mL of Proteinase K for an additional 2.5 hours at 37°C. Following electrophoresis, slides were air dried over night and then stained in Sybr- Green before microscopic analysis. Only sperm with clearly extended Comet tails (at least 2-fold greater than the average comet tail size) were scored as positive.

### Apoptosis Analysis

Whole testis were isolated, fixed in 4% paraformaldehyde/PBS solution at 4C over-night solution. Six micrometer thick sections were mounted on silanized glass slides and processed using the Apoptag Plus Peroxidase Apoptosis Detection Kit (Chemicon, Temecula, CA) according to manufacturers' protocol.

### TRAP assay

Testis were snap frozen in liquid nitrogen and then homogenized using mortar and pestle. Telomerase extracts were prepared using CHAPS lysis buffer, and protein concentration was determined using the Bradford assay. The TRAP assay was performed as previously described [26] using 5ug from each sample testis extract.

### RNA Isolation and Labeling for Array Analysis

Whole testis were snap frozen in liquid nitrogen, ground with mortar and pestle, followed by extraction of total RNA with Trizol reagent. Quality of RNA for all samples was assessed on 1% agarose gels. Labeled cDNA was generated using the MicroMax Direct Labeling kit (Perkin Elmer). Briefly, 20ug of each RNA sample was used as template in reverse transcription (RT) reactions using an oligo dT primer. Each RT reaction included either Cy3 or Cy5 conjugated nucleotides to label the cDNA product. Input RNA was hydrolyzed after each RT reaction using NaOH, and cDNA was purified by isopropanol precipitation. The cDNA samples from sibling Sirt1−/− and Sirt1+/− mice (each labeled with a different fluorophor) were then washed briefly, re-suspended in hybridization buffer (see below), combined together, and immediately used in the hybridization step.

### cDNA Hybridization and Data Analysis

After a brief rinse in 1× SSC buffer, each array slide was immersed in pre-hybridization buffer (3× SSC, 25% formamide, 0.2% BSA and 0.1% SDS) for 30 minutes at 42C. Slides were then rinsed in water, then 95% ethanol, and finally spun dry prior to hybridization. The labeled cDNA probe mixture, re-suspended in hybridization buffer (3× SSC, 25% formamide, 0.1% SDS, 0.3ug/ul yeast tRNA), was heated to 95C for 2 minutes just prior to applying to the slide. All hybridizations were performed at 42C over night in sealed hybridization chambers immersed in a water bath. After hybridization, slides were washed in the following solutions, in order, for 15 minutes each at room temperature- 0.5× SSC, 0.01%SDS, 0.06× SSC, 0.01% SDS, 0.06× SSC.

After the final wash step, slides were immediately scanned using a GenePix 4000b array scanner and GenePix Pro 6.0 software, at settings which produced equivalent signal in both Cy3 and Cy5 channels and low background. Array data was then transferred to Acuity 4.0 software for normalization and analysis.

### Quantitative RT-PCR

Real time RT PCR was performed using a Roche Light-Cycler, as previously described [23] with the except that 1ug of total RNA from pooled Sirt1−/− or Sirt1+/− testis RNA samples (equal amounts of total RNA used from each testis RNA sample to derive the Sirt1−/− and Sirt1+/− RNA pools) was used in the RT reactions. All primer sets used in the PCR step were designed to span a terminal intron and produce an amplicon in the size range of 100–150 bp. The production of a single PCR product was also verified by gel electrophoresis for each primer set.

## Results

### Sirt1 deficiency abrogates spermatogenesis

**Figure 1:**
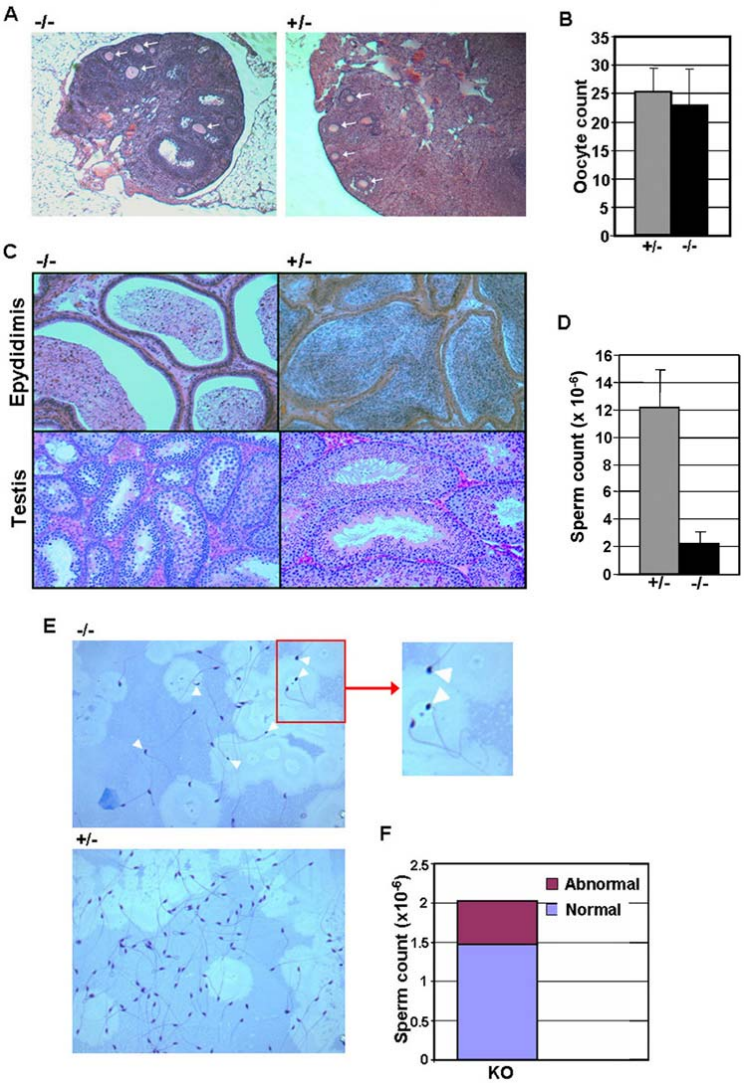
Sirt1 deficiency effects spermatogenesis, but not oogenesis. A. Cross section of ovaries from sibling female Sirt1+/− and Sirt1−/− mice following superovulation. Mature oocytes are indicated by arrowheads. B. Average number of oocytes for female Sirt1+/− and Sirt1−/− mice (n = 10; P>0.1). C. Cross section of testis and epididymis from sibling male Sirt1+/− and Sirt1−/− mice. D. Average number of sperm from the cauda epididymis for male Sirt1+/− and Sirt1−/− mice (n = 3 sibling pairs). P values for comparison of average counts (student's t Test) are shown. E. Analysis of sperm morphology. Total epididymal sperm were fixed to slides and stained with Diff-Quik staining media. Sperm with misshapen heads are indicated by arrowheads. F. Frequency of abnormal sperm from epididymis of Sirt1−/− mice. The number of abnormal sperm for Sirt1+/− mice was <<1%. In all analyses, mice were 8 weeks of age.

To assess the affect of Sirt1 deficiency on germ cell development, we initially examined germ cell number in sibling Sirt1−/− and Sirt1+/− male and female mice. As shown in figure 1, histological analysis of ovaries from female mice 9 hours after hCG injection revealed no substantial deficit in oocyte maturation in Sirt1−/− females. In addition, no difference in the average number of mature oocytes produced following superovulation was observed between Sirt1−/− and Sirt1+/− female mice (Fig 1A,B), confirming the lack of effect of Sirt1 deficiency on oocyte production. In contrast, histological analysis of testis and cauda epididymis from Sirt1−/− and Sirt1+/− males showed reduced number of sperm, as well as small and abnormal seminiferous tubules (Fig 1C). Furthermore, the average epididymal sperm count was reduced ∼10 fold in Sirt−/− mice (Fig 1D). The frequency of abnormal sperm, for example sperm with misshapen heads or sperm lacking a tail, was also elevated in Sirt1−/− mice (Fig 1E,F), in agreement with previous observations^18^. In addition, analysis of the frequency of apoptotic cells in the testis of Sirt1−/− and Sirt1+/− mice was elevated (Figure S1). Together, these results show that Sirt1 deficiency abrogates spermatogenesis, but not oogenesis.

We and others [18] have found that male and female Sirt1−/− mice fail to reproduce when paired with wild type mice (6 out of 6 mating pairs for both male and female Sirt1−/− mice failed to produce litters when paired with B6D2F1 mice). To assess the effect of Sirt1 deficiency on germ cell function in more detail, we utilized male and female gametes from Sirt1−/− and Sirt1+/− mice in *in vitro* fertilization (IVF) experiments to examine the relative efficiency of developing zygotes as well as development to term following embryo transfer to surrogate mothers. We find that both sperm and oocytes from Sirt1−/− mice are capable of fertilization, although the efficiency of developing to the 2-cell stage is substantially compromised for both sperm and oocytes from Sirt1−/− mice as compared to gametes from Sirt1+/− mice (Table 1). Nevertheless, we were able to reproducibly obtain live pups when performing IVF with Sirt1−/− sperm or oocytes together with gametes from B6D2F1 mice. Furthermore, the F1 Sirt1+/− mice from these crosses were fertile, and had a general phenotype indistinguishable from that of Sirt1+/− or Sirt1+/+ mice. When performing IVF with both Sirt1−/− sperm and oocytes, we are also able to obtain viable F1 Sirt1−/− pups. These F1 Sirt1−/− pups are also capable of surviving to adulthood, and appear to have a similar phenotype to Sirt1−/− mice generated by breeding Sirt1+/− mice.

**Table 1:** Analysis of Germ Cell Function using In Vitro Fertilization

*Sperm* 1	*Oocytes* 1	*Oocytes #*	*2-Cell (%)*	*Blastocyst (%)*	*# embryos Transferred* 3	*Live Births (%)*
BDF1	BDF1	254	223 (88)^2^	102 (83)	16	8 (50)^4^
BDF1	HET	117	104 (89)	61 (59)	0	ND
HET	BDF1	102	78 (76)	5 (100)	0	ND
BDF1	KO	91	61 (67)	43 (70)	32	6 (19)
KO	BDF1	32	19 (59)	16 (84)	16	4 (25)
HET	HET	45	34 (76)	ND	34	11 (32)
HET	KO	27	8 (30)	ND	0	ND
KO	HET	76	28 (37)	ND	0	ND
KO	KO	120	41 (34)	38 (93)	38	5 (13)

1- All mice were 8–9 weeks of age.

2- Numbers in parentheses represent the percentage of 2-cell embryos as a function of the number of oocytes fertilized

3- Embryos were transferred at the 2-cell stage for all IVF experiments. Embryos were transferred to pseudo-pregnant mothers for some but not all IVF experiments.

4- The number in parentheses represents the percentage of live birth as a function of the number of embryos transferred.

### Sirt1 deficiency effects male gametogenesis during development

**Figure 2:**
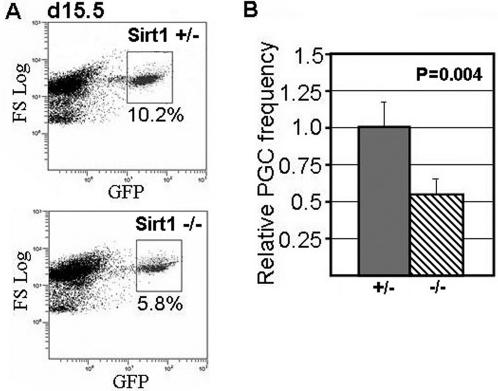
Spermatogenic stem cell numbers are reduced in Sirt1 deficient embryos. A. Sirt1+/− mice were back-crossed with Oct4-GFP transgenic mice to generate Sirt1+/− mice that were homozygous for the Oct4-GFP transgene. These mice were then bred, and gonads were isolated from d15.5 male embryos. Single cell suspensions were prepared for each gonad sample, and the frequency of GFP+ cells was assessed using FACS (see Materials and Methods and reference 23 for further details). The genotype of each sample was confirmed retrospectively using PCR. Sample FACS plots for a Sirt1−/− and sibling Sirt1+/− male embryo are shown. B. Average frequency of GFP+ cells per embryo for Sirt1+/− or Sirt1+/− or Sirt1+/+ embryos (Sirt1−/−, n = 4; Sirt1+/− and Sirt1+/+, n = 8). Note, no significant difference in GFP+ frequency was observed for Sirt1+/− and Sirt1+/+ mice (data not shown).

We and others have previously shown that the Oct4-GFP transgene is a useful marker for the identification and purification of primitive germ stem cells from mouse embryos [22], [23]. Thus, to examine the effect of Sirt1 deficiency on early spermatogenic precursors during development, we crossed the Sirt1 knock-out strain with the Oct4-GFP transgenic strain to allow identification and purification of spermatogenic stem cells from embryonic gonads. In d15.5 male embryos, we find that spermatogenic stem cells (ie. d15.5 pro-spermatogonia cells) are reduced in numbers by ∼50% in Sirt1 deficient embryos as compared to sibling Sirt1+/− embryos (Fig 2). These data show that the effect of Sirt1 on male germ cells is evident at least as early as d15.5 of development.

### Sirt1 deficiency induces elevated DNA damage in male germ cells

Since Sirt1 has been implicated in the regulation of expression of telomerase reverse transcriptase (Tert) [27] we examined telomerase activity levels in Sirt1−/− and Sirt1+/− testis. We did not observe any appreciable effect of Sirt1 deficiency on telomerase activity levels in the testis (Figure S2), indicating that accelerated telomere attrition is unlikely to have a major role in the effect of Sirt1 deficiency in male germ cells.

**Figure 3:**
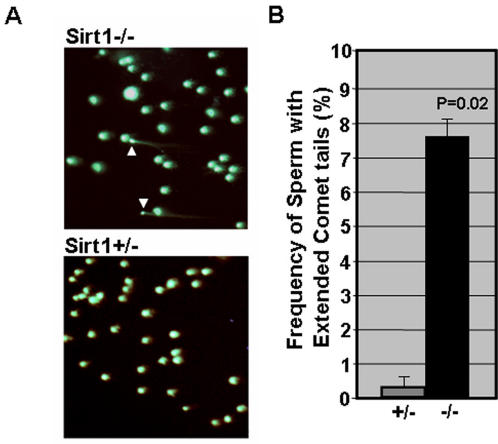
DNA damage is elevated in sperm from Sirt1 deficient mice. A. Image of sperm nuclei from Sirt1+/− and Sirt1−/− mice stained with Sybr-Green following single cell alkaline electrophoresis (Comet assay). The Comet assay was performed using the Trevigen comet assay kit. Sperm with extended comet tails are indicated with arrowheads. B. Frequency of sperm with extended Comet tails for pairs of sibling Sirt1+/− and Sirt1−/− mice (n = 2).

Recent studies have implicated that Sirt1 may play a role in maintaining genomic integrity [16], [28] in mammalian cells. Therefore, we sought to compare the status of genomic integrity in sperm from Sirt1−/− and Sirt1+/− mice using the Comet assay (single cell alkaline electrophoresis). As shown in figure 3, we observed very few sperm (<1%) with extended comet tails in Sirt1+/− mice, whereas approximately ∼8% of sperm from Sirt1−/− mice had extended comet tails. These results show that the number of sperm with DNA damage, specifically, the number of sperm with single or double strand DNA breaks, is significantly elevated in Sirt1 deficient mice.

### Sirt1 deficiency effects expression of genes involved in spermatogenesis

**Figure 4:**
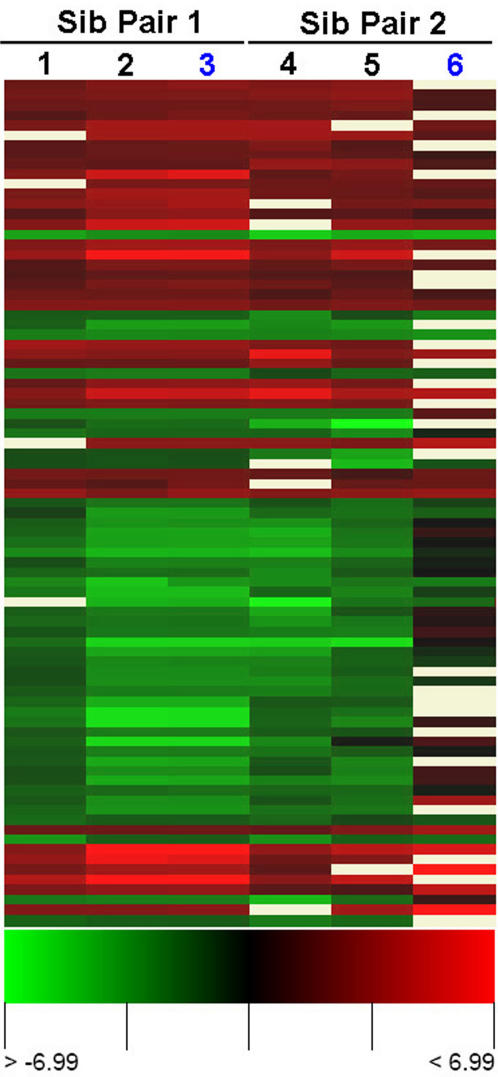
Heat map representation of differentially expressed genes in the testis of Sirt1 deficient mice. Rows represent genes and columns represent array comparisons of Sirt1−/− and Sirt1+/− RNA samples as indicated. Green indicates genes that are up-regulated in the Sirt1−/− testis samples. Columns labeled in blue are analyses done using dye reversal. Only differentially expressed genes showing an average of 1.7 or greater fold change in expression across at least 5 of the 6 arrays analyzed are included in this analysis.

**Figure 5:**
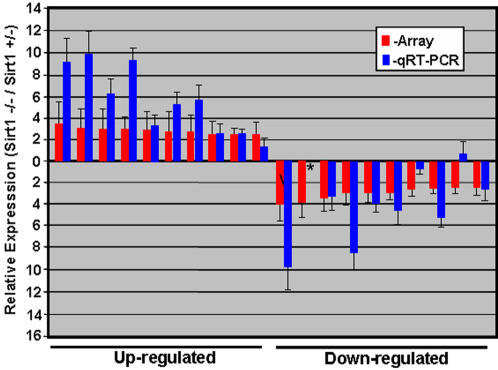
Quantitative RT-PCR analysis of the expression of the top 10 up and down regulated genes in the testis of Sirt1 deficient mice. The relative expression level of the top 10 differentially expressed genes, both up and down regulated (see Suppl. Table S1), was assessed using real-time RT-PCR for the Sirt1−/− and Sirt1+/− RNA samples. All PCR primers were designed to flank the most 3′ intron, and were shown to yield a single PCR product in the size range of 100–150 bp. Each analysis was performed intriplicate, and error bars representing standard deviation are shown. Expression level for qRT-PCR analysis is shown relative to the level of expression of Hprt.

To assess the effect of Sirt1 deficiency on spermatogenesis in more detail, we compared global gene expression for Sirt1−/− and Sirt1+/− testis using spotted oligonucleotide microarray technology (all analyses were performed using the MEEBO oligonucleotide library- microarrays were purchased from Stanford University microarray facility). In this analysis, we performed intriplicate analysis of global gene expression, including dye swap analysis, for 2 pairs of sibling Sirt1−/− and Sirt1+/− mice. We identified a total of 85 differentially expressed genes, with a >1.7 fold change in gene expression, either up or down, in at least 5 of the 6 arrays analyzed (Fig 4; the microarray data from this study may be accessed at the GEO database with accession number (GSE8492)). Analysis of the relative mRNA levels for the top 10 up and down regulated genes (see Suppl Table S1) by qRT-PCR confirmed the differential expression of all these genes (Fig 5), and showed an overall concordance in relative expression levels with that obtained from microarray analysis.

To objectively assess which genes were differentially regulated, we used the gene classification tool Gene Ontology (GO) [29]. Gene ontology analysis using GOstat revealed an over-representation of differentially expressed genes in the GO category spermatogenesis ((Hook1, RNF17, Nasp, Spesp1, and Dnaja1). Interestingly, genes involved in sumoylation of proteins (SUMO1 and SUMO2) were also over-represented. For both the genes involved in spermatogenesis and sumoylation, over-expression was observed in the testis of Sirt1−/− mice (see Discussion section).

## Discussion

Sirt1 has been shown to have a significant role in the regulation of glucose metabolism and the promotion neural cell survival. In this study, we have examined in detail the role of Sirt1 in germ cell function and gametogenesis using a Sirt1 knock-out mouse strain. We find that both mature sperm and spermatogenic stem cell numbers are significantly reduced in Sirt1 deficient mice and embryos respectively, whereas oocyte numbers are not affected. While the fertility of Sirt1 deficient gametes is markedly attenuated, we were able to generate F1 Sirt1−/− mice which survived to adulthood by performing IVF with gametes from Sirt1−/− mice. The deleterious effect of Sirt1 deficiency on sperm numbers is at least in part accounted for by elevated levels of DNA damage, as assessed using the Comet assay. Microarray analysis of global gene expression in the testis from Sirt1 deficient mice revealed aberrant expression of a number of genes involved in spermatogenesis. Together, these data demonstrate that Sirt1 has important roles in spermatogenesis and germ cell function.

While we did not observe an affect of Sirt1 deficiency on the ability to produce mature oocytes, we did notice a marked decrease in efficiency in generating both 2-cell embryos and live-offspring when performing IVF with oocytes from Sirt1−/− females (Table S1). We have confirmed that Sirt1 is expressed in oocytes (data not shown), and therefore, these results suggest Sirt1 may play a role in fertilization and/or early stages of embryogenesis. Nevertheless, the ability to generate live offspring utilizing gametes from both Sirt1−/− males and females (Table 1 and reference 20) clearly shows that Sirt1 is not essential for embryogenesis. Whether the survival of these F1 Sirt1 deficient embryos is due to compensation by other Sirtuins or other proteins remains to be assessed.

The observation that Sirt1 deficiency causes a reduction not only in the numbers of mature sperm in adult mice (Fig 1) but also in numbers of spermatogenic stem cells(Fig 2) from d15.5 male embryos indicates that Sirt1 may have important roles in certain types stem cells. Based on recent observations by us and others, we predict that the exact nature of the effect of Sirt1 deficiency will likely be lineage dependent. For example, while Sirt1 deficiency attenuates spermatogenesis, mouse embryonic fibroblasts deficient in Sirt1 exhibit enhanced survival in response to genotoxic stress and the ability to bypass cell senescence [21]. Furthermore, we have observed enhanced survival and growth potential of HSC from Sirt1 deficient mice during culture in cytokine deprived media, and negligible differences in numbers of HSC between young Sirt1−/− and Sirt1+/− mice (unpublished observations). Thus Sirt1 likely has different functions in different cell types, including stem cells.

Global analysis of gene expression revealed an over-representation of genes in the GO categories of spermatogenesis and protein sumoylation. The former observation is in agreement with the reduced sperm count and increased frequency of abnormal sperm and DNA damage in sperm from Sirt1 deficient mice. Most of the differentially expressed genes involved in spermatogenesis were over-expressed in the testis of Sirt1 deficient mice. Sirt1 has the potential to promote transcriptional silencing, for example by deacetylating and inactivating the transcription factors p300 and FOXO proteins [30], [31]. Therefore, the effect of Sirt1 deficiency on expression of genes involved in spermatogenesis in the testis could be a direct affect at the level of transcription. However, the expression of a number of these genes occurs predominantly at specific stages of spermatogenesis. For example, Hook1 is expressed predominantly in spermatocytes and round spermatids [32] and Nasp is predominantly expressed in spermatocytes [33]. Thus a possible affect due to altered cellularity, the relative numbers of different types of spermatogenic precursors, in the testis of Sirt1 deficient mice on differential expression of these genes cannot be presently ruled out. It will be of interest to assess whether Sirt1 regulates transcription of genes involved in spermatogenesis and/or alters the proportions of spermatogenic cells in the testis, perhaps by causing a block at a specific stage of spermatogenesis, in future studies.

Interestingly, genes involved in the sumoylation of proteins, the addition or removal of small ubiquitin-like modifiers (SUMO) to proteins, are also over-represented in the genes differentially expressed in Sirt1 deficient testis. Recent studies have suggest that Sirt1 can affect the sumoylation of certain proteins via a mechanism where the deacetylation of specific Lys residues by Sirt1 allows the subsequent addition of SUMO by other proteins [30], [34]. It is possible that, the over-expression of SUMO-1 and SUMO-2, both of which are capable of adding SUMO to other proteins, in the testis of Sirt1 deficient mice may be a compensatory response to promote the sumoylation of specific proteins in the testis in the absence of the deacetylase activity of Sirt1. The exact role of Sirt1 in the regulation of sumoylation of proteins in spermatogenic cells, and the relevance of this to spermatogenesis, remains to be addressed.

Results from recent studies on the ability of Sirt1−/− mice to successfully mate and produce offspring with other mice are incongruent. A study by McBurney et al [18] and the results presented here suggest that Sirt1−/− mice are infertile, in that they cannot successfully mate with other mice, are at least that fertility is severely compromised by Sirt1 deficiency. However, Gu et al have recently reported that Sirt1−/− mice, both males and females, can generate offspring by mating with wild-type mice [20]. While the reason for this discrepancy is not presently understood, it is unlikely to be accounted for by the expression of different dysfunctional versions of Sirt1 in the different Sirt1 knock-out strains, since both the strain used by Gu et al [20] and that used in our study were derived from the same Sirt1 knock-out ES cell strain (Exon 4 of the Sirt1 gene is deleted in both strains) [19]. One possible explanation is hybrid vigor, since the Sirt1−/− strain used by Gu et al was on a 129SvJ/C57BL6 background [20] whereas the strain used by McBurney et al was on a 129/J background, and the Sirt1−/− mice used here had been back-crossed onto a C57BL6 background. Nevertheless, our ability to generate viable offspring using IVF with Sirt1−/− gametes, in both Sirt1−/− X wild type crosses and Sirt1−/− X Sirt1−/− crosses (Table S1), shows that Sirt1−/− mice (at least those derived from Sirt1+/− X Sirt1+/− crosses) are not sterile, in agreement with the results of Gu et al. Furthermore, the observation that viable F2 Sirt1−/− mice may be readily generated using IVF (Table S1) shows that assisted reproductive technologies may be a practical method to study the long term effect of Sirt1 deficiency, across multiple generations of Sirt1−/− mice, on development and aging.

## Supporting Information

Figure S1 Numbers of apoptotic cells is increased in Sirt1 testis. Testis from Sirt1+/− and Sirt1−/− mice were dissected, fixed, embedded in paraffin, and sectioned. The sections were then stained for apoptotic cells using the ApoTag kit (Chemicon- CHECK). A. Sample images of stained sections from Sirt1−/− and Sirt1+/− mice are shown. Apoptotic cells are indicated by arrowheads. B. Quantitative analysis of the number of apoptotic cells per seminiferous tubule. The number of tubules in Sirt1−/− testis with no detectable apoptotic cells was <1%.
(3.16 MB TIF)

Figure S2 Telomerase activity is not affected by Sirt1 deficiency in the testis. Extracts were prepared from testis from Sirt1−/− and Sirt1+/− mice and telomerase activity was measured using the TRAP assay according to manufacturers' protocol (TRAPeze Kit; Chemicon). A. Sample blot showing TRAP assay results for testis samples from sibling Sirt1−/− and Sirt1+/− mice. The internal control PCR product is indicated by the arrowhead. B. Quantitative analysis of telomerase activity. The level of telomerase activity was assessed for Sirt1−/− and Sirt1+/− testis samples according to manufacturers' protocol. The bars represent average results from intriplicate analyses of testis samples from 3 pairs of sibling Sirt1−/− and Sirt1+/− mice (all 8 weeks of age). Error bars representing standard deviation are shown.
(0.65 MB TIF)

Table S1 Top 10 up and down regulated differentially expressed genes in testis
(0.03 MB DOC)
