# Supplementary figures and images for: Sirt1 Deficiency Attenuates Spermatogenesis and Germ Cell Function
Source: PLoS One. 2008 Feb 13;3(2):e1571. doi: 10.1371/journal.pone.0001571 (PMC2216432; doi:10.1371/journal.pone.0001571)

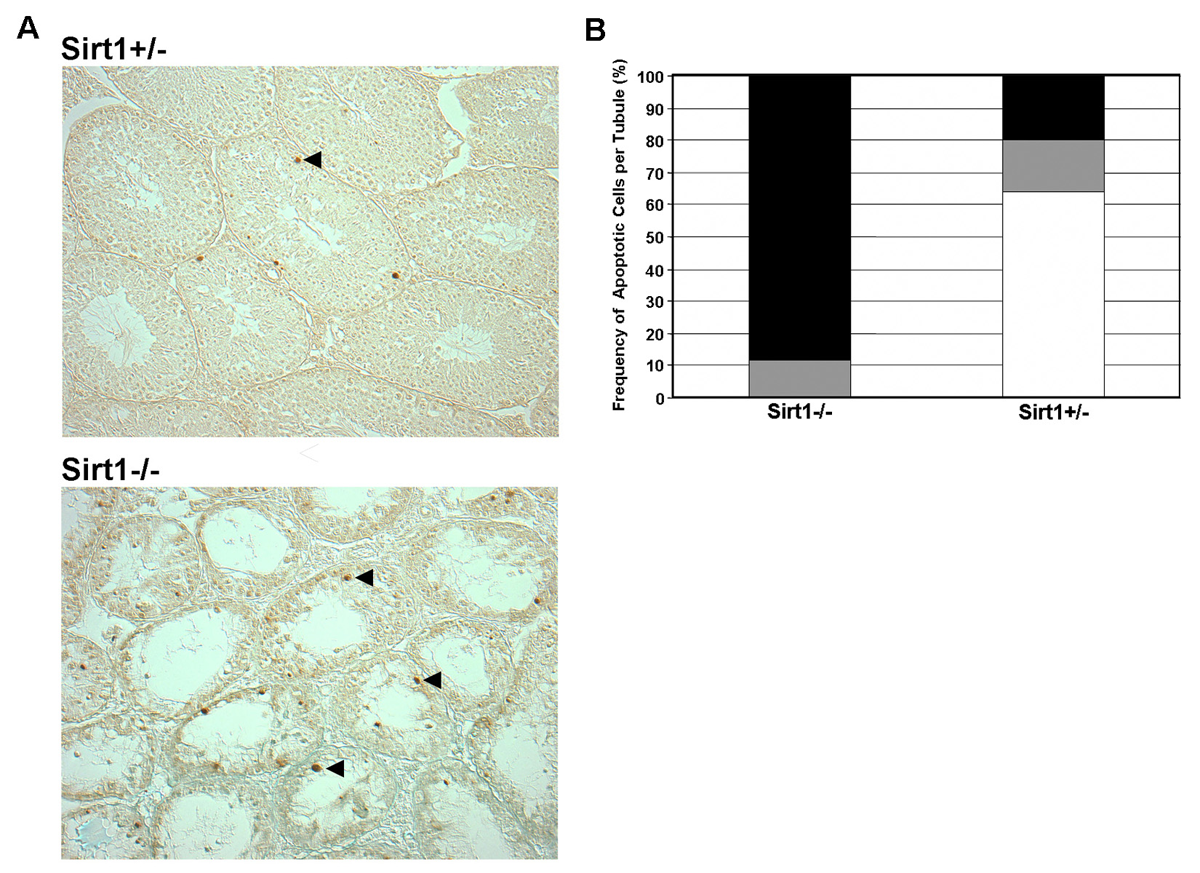

Supplement: Figure S1 — Numbers of apoptotic cells is increased in Sirt1 testis. Testis from Sirt1+/− and Sirt1−/− mice were dissected, fixed, embedded in paraffin, and sectioned. The sections were then stained for apoptotic cells using the ApoTag kit (Chemicon- CHECK). A. Sample images of stained sections from Sirt1−/− and Sirt1+/− mice are shown. Apoptotic cells are indicated by arrowheads. B. Quantitative analysis of the number of apoptotic cells per seminiferous tubule. The number of tubules in Sirt1−/− testis with no detectable apoptotic cells was <1%. (3.16 MB TIF) [file pone.0001571.s001.tif]

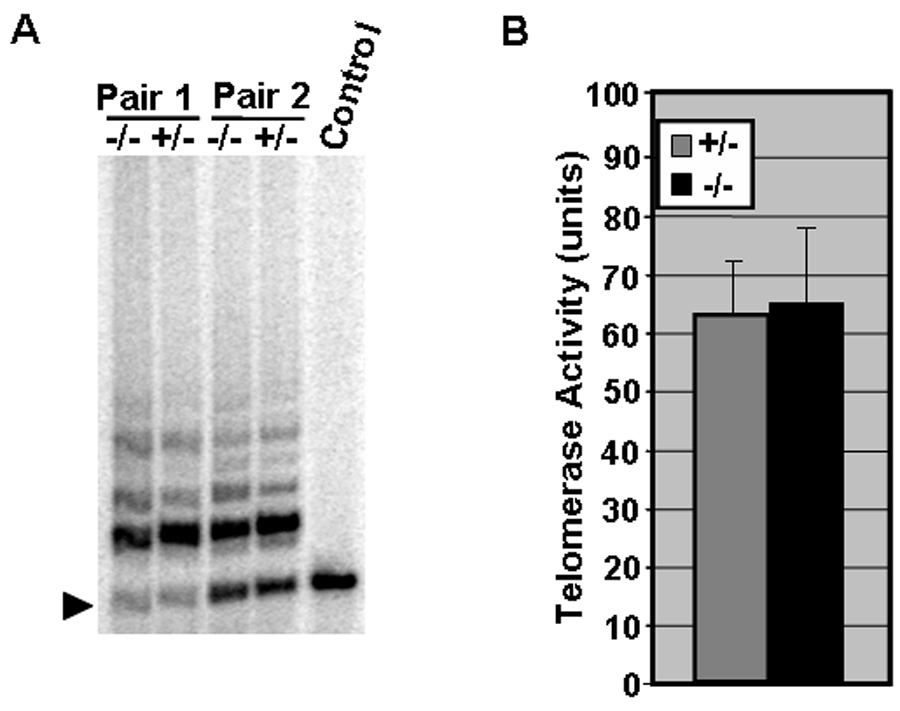

Supplement: Figure S2 — Telomerase activity is not affected by Sirt1 deficiency in the testis. Extracts were prepared from testis from Sirt1−/− and Sirt1+/− mice and telomerase activity was measured using the TRAP assay according to manufacturers' protocol (TRAPeze Kit; Chemicon). A. Sample blot showing TRAP assay results for testis samples from sibling Sirt1−/− and Sirt1+/− mice. The internal control PCR product is indicated by the arrowhead. B. Quantitative analysis of telomerase activity. The level of telomerase activity was assessed for Sirt1−/− and Sirt1+/− testis samples according to manufacturers' protocol. The bars represent average results from intriplicate analyses of testis samples from 3 pairs of sibling Sirt1−/− and Sirt1+/− mice (all 8 weeks of age). Error bars representing standard deviation are shown. (0.65 MB TIF) [file pone.0001571.s002.tif]
