# Supplementary material for: Sirt1 Deficiency Attenuates Spermatogenesis and Germ Cell Function
Source: PLoS One. 2008 Feb 13;3(2):e1571. doi: 10.1371/journal.pone.0001571 (PMC2216432; doi:10.1371/journal.pone.0001571)
Supplement: Table S1 — Top 10 up and down regulated differentially expressed genes in testis (0.03 MB DOC) [file pone.0001571.s003.doc]

**UPREGULATED**

**Gene Function Fold change**

**1. Kif3a kinesin family member 3A 3.5<2>†**

**2. Eif5b eukaryotic translation initiation factor 5B 3.1**

**3. Vwc2 von Willebrand factor C domain containing 2 3.0**

**4. Hook1 hook1 homolog (Drosophila); abnormal spermatozoon head shape 2.9<2>**

**5. Nasp mus musculus nuclear autoantigenic sperm protein (histone-binding) 2.9**

**6. Sucla2 succinate-Coenzyme A ligase, ADP-forming, β-subunit 2.7**

**7. Prelp proline/arginine-rich end leucine-rich repeat protein 2.7**

**8. Khdrbs1 KH domain containing, RNA binding, signal transduction associated 1 2.5**

**9. Dnaja1 mus musculus DnaJ (Hsp40) homolog, subfamily A, member 1 2.5<2>**

**10. Nsbp1 nucleosome binding protein 1 2.4**

**DOWN-REGULATED**

**Gene Function Fold change**

**1. Spesp1 sperm equatorial segment protein 1 4.0**

**2. SLC9a1 solute carrier family 22 (anion transporter) 1 3.9**

**3. SPIN-2 spindlin-like protein 2 3.6**

**4. Strn4 calmodulin binding protein 4 3.0**

**5. Mgst2 microsomal glutathione S-transferase 2 3.0**

**6. Tmod4 mus musculus tropomodulin 4 2.9 <2>**

**7. Interleukin3 cytokine 2.6**

**8. Gfer growth factor, erv1 (S. cerevisiae)-like (augmenter of liver regeneration) 2.5**

**9. Slc22a7 solute carrier family 22 (anion transporter) 7 2.4**

**10. Klhl8 kelch-like 8 (Drosophila) 2.3**

**10. FXyd5 FXYD domain-containing ion transport regulator 5 2.3 <2>**

†- Number in parentheses is included for genes represented on the array by more than 1 feature, and represents the number of features for that gene yielding a consistent pattern of differential expression across 5 or more arrays.
